# Overexpressed TPX2 causes ectopic formation of microtubular arrays in the nuclei of acentrosomal plant cells

**DOI:** 10.1093/jxb/ert271

**Published:** 2013-09-04

**Authors:** Beáta Petrovská, Hana Jeřábková, Lucie Kohoutová, Věra Cenklová, Žaneta Pochylová, Zuzana Gelová, Gabriela Kočárová, Lenka Váchová, Michaela Kurejová, Eva Tomaštíková, Pavla Binarová

**Affiliations:** ^1^Centre of the Region Haná for Biotechnological and Agricultural Research, Institute of Experimental Botany, AS CR, v.v.i., Šlechtitelů 31, Olomouc 783 71, Czech Republic; ^2^Institute of Microbiology, AS CR, v.v.i., Vídeňská 1083, 142 20 Prague 4, Czech Republic; ^3^Institute of Experimental Botany, AS CR, v.v.i., Sokolovská 6, 772 00 Olomouc, Czech Republic

**Keywords:** Arabidopsis thaliana, AtTPX2, Aurora kinase, fibres, γ-tubulin, importin, microtubules, nuclei, Ran.

## Abstract

TPX2 performs multiple roles in microtubule organization. Previously, it was shown that plant AtTPX2 binds AtAurora1 kinase and colocalizes with microtubules in a cell cycle-specific manner. To elucidate the function of TPX2 further, this work analysed *Arabidopsis* cells overexpressing AtTPX2-GFP. Distinct arrays of bundled microtubules, decorated with AtTPX2-GFP, were formed in the vicinity of the nuclear envelope and in the nuclei of overexpressing cells. The microtubular arrays showed reduced sensitivity to anti-microtubular drugs. TPX2-mediated formation of nuclear/perinuclear microtubular arrays was not specific for the transition to mitosis and occurred independently of Aurora kinase. The fibres were not observed in cells with detectable programmed cell death and, in this respect, they differed from TPX2-dependent microtubular assemblies functioning in mammalian apoptosis. Colocalization and co-purification data confirmed the interaction of importin with AtTPX2-GFP. In cells with nuclear foci of overexpressed AtTPX2-GFP, strong nuclear signals for Ran and importin diminished when microtubular arrays were assembled. This observation suggests that TPX2-mediated microtubule formation might be triggered by a Ran cycle. Collectively, the data suggest that in the acentrosomal plant cell, in conjunction with importin, overexpressed AtTPX2 reinforces microtubule formation in the vicinity of chromatin and the nuclear envelope.

## Introduction

The targeting protein for Xklp2 (TPX2), is a microtubule-associated protein with multiple functions. Originally, it was identified as a protein required for targeting kinesin-12 (Xklp2) to the spindle pole in *Xenopus* egg extracts (Wittmann *et al.*, 1998). TPX2 and NuMA have been identified as potential downstream effectors of RanGTP in microtubule assembly in *Xenopus* egg extracts and both proteins are the targets of importin blocking activity; they are found in complexes with importin α and β (Gruss *et al.*, 2001; Nachury *et al.*, 2001; Wiese *et al.*, 2001). TPX2 and NuMA proteins colocalize to the interphase nucleus, probably with Ran and importin α and β. This nuclear localization prevents them from acting on microtubules in the cytoplasm until the nuclear envelope breaks down at the beginning of mitosis or meiosis (Kahana and Cleveland, 2001). In the initial phase of mitosis, RanGTP releases TPX2 from its interphase binding partner, importin β, and thus activates TPX2 for bipolar spindle assembly.

TPX2 is also a well-characterized upstream regulator of Aurora A kinase (Kufer *et al.*, 2002; Eyers *et al.*, 2003; Marumoto *et al.*, 2005) and TPX2-activated Aurora A kinase was shown to be essential for Ran-stimulated spindle assembly in the presence/absence of centrosomes (Tsai and Zheng, 2005). TPX2 is an important protein in centrosomal and acentrosomal microtubule nucleation in chromatin and its role with γ-tubulin in chromatin-driven mitotic spindle nucleation in animal cells is well characterized (Wilde and Zheng, 1999; Groen *et al.*, 2004). Recently, TPX2 was identified as a new scaffolding protein and a co-activator of Aurora B in the chromosomal passenger complex (Iyer and Tsai, 2012). Proteomic analysis of human metaphase chromosomes has shown that TPX2 is a nuclear protein belonging to the group of chromosomal fibrous proteins (Uchiyama *et al.*, 2005). However, of the 18 different proteins in this group (e.g. β-actin, vimentin, tubulin), the contribution of TPX2 is unknown (Pederson and Aebi, 2002).

Plant TPX2 contains all of the functional domains of its vertebrate counterpart, but the TPX2 signature motif is present only once in vertebrate sequences compared to twice in plants (Vos *et al.*, 2008; Evrard *et al.*, 2009) where its coiled-coil signature is poorly understood. In *Arabidopsis*, two copies of the TPX2 gene are expressed per genome (Vos *et al.*, 2008), where it is predominantly nuclear during interphase but is actively exported before nuclear envelope breakdown. AtTPX2 is essential for nuclear envelope breakdown and initiation of prospindle assembly (Vos *et al.*, 2008; Evrard *et al.*, 2009). Plant microtubule-associated proteins sharing the same microtubule binding domain as TPX2 play important roles in the organization of microtubular arrays, cell growth, and regulation of cell division (for reviews see Hamada, 2007; Sedbrook and Kaloriti, 2008). Recently, Panteris and Adamakis (2012) speculated about the possible role of fern TPX2 in cortical microtubule assembly.

In 2012, this study group reported that AtAurora1 kinase and AtTPX2 colocalize in plant microtubules in a cell cycle-specific manner, from preprophase to early telophase (Petrovská *et al.*, 2012). In addition, *Arabidopsis* TPX2 protein is intranuclear, and although important mitotic functions for the protein have already been well documented (Vos *et al.*, 2008; Petrovská *et al.*, 2012), any functional role for its accumulation in interphase nuclei is far from being understood.

This study presents data on specific arrays of microtubules decorated with AtTPX2 formed in nuclei and in the vicinity of the nuclear envelope of cells overexpressing AtTPX2-GFP. The formation of nuclear and perinuclear microtubules occurred without participation of Aurora kinase 1 and mitotic signalling. Microtubular arrays heavily decorated with AtTPX2 were not specific to programmed cell death as was described in mammalian cells (Moss *et al.*, 2009). These data on the functions of AtTPX2 in the formation of specific nuclear/perinuclear microtubular arrays and the interaction of importin with AtTPX2 bring further insight to the poorly understood molecular mechanisms of acentrosomal plant microtubule organization.

## Materials and methods

### Molecular cloning of *AtTPX2* and *AtAurora1*

Molecular cloning of *AtTPX2* (At1g03780) and *AtAurora1* (At4g32830) for N- and C-terminal fusions was performed according to Petrovská *et al*. (2012). Gateway binary vectors pK7WGF2,0 for N-terminal GFP fusion, pH7WGR2,0 for N-terminal RFP fusion (Karimi *et al.*, 2002), pMDC43 for C-terminal GFP fusion (Curtis and Grossniklaus, 2003), and pB7RWG2,0 for C-terminal RFP fusion (Karimi *et al.*, 2002) for *AtTPX2* cloning were used, and pGEM T-Easy P2R-P3 (Invitrogen), pGEM T-Easy P4-P1R (Invitrogen), pGEM T-Easy 221 (Invitrogen), and pK7m34GW (purchased from Ghent University, Ghent, Belgium) for *AtAurora1* cloning were used.

### Stable transformation of cell suspension cultures and plants

Suspension cultures of *Arabidopsis thaliana* cv. Columbia and cv. Lansberg erecta (Ler) with stable expression of AtTPX2-GFP or/and AtAurora1-RFP were derived as described in Petrovská *et al*. (2012), using the techniques of Mathur *et al.* (1998) and Koroleva *et al.* (2005). *Arabidopsis* Columbia plants were transformed with AtTPX2-GFP using the floral-dip method (Clough and Bent, 1998) as described in Petrovská *et al*. (2012).

### Quantitative real-time PCR analysis

Quantitative real-time PCR (qPCR) was performed following MIQE recommendations (Bustin *et al.*, 2009). Total RNA was isolated from *A. thaliana* control and AtTPX2-overexpressed suspension cultures using the Plant RNeasy Extraction Kit (Qiagen). Digestion of DNA during RNA purification was performed using the RNase-Free DNase Set (Qiagen). Purified RNA (100ng) was reverse transcribed using the Transcriptor High Fidelity cDNA Synthesis Kit (Roche) with an anchored-oligo (dT)_18_ primer according to the Roche instructions. *QPDR* using SYBR Green I Dye (Top-Bio, Czech Republic) was performed using the CFX96 Real-Time PCR Detection System (Bio-Rad). Three replicate PCR amplifications were performed for each sample. The PDF2 gene (Czechowski *et al.*, 2005) was used as a reference. Quantification of transcripts of each gene, normalized to the internal reference PDF2 gene (At1g13320), was determined using CFX Manager Software (Bio-Rad). The transcript level of each target gene of control cells or the reference gene in controls or overexpressed AtTPX2 cells, was designated as 1.0. The primers used for real-time PCR were: PDF2For 5′-TAACGTGGCCAAAATGATGC-3′, PDF2Rev 5′-GTTCTCCACAACCGCTTGGT-3′, *AtTPX2*For 5′-AAGCTCGACCTGTGAACAAGA-3′, and *AtTPX2*Rev 5′-CTGGCAGATGTGGTGTACTTCT-3′. To ensure specificity of primers, primer pairs were designed to span across two neighbouring exons and were detected as a single peak in dissociation curve analysis.

### Drug treatment

Amiprophos methyl (APM; Duchefa) at a concentration of 5 µM was used for microtubule depolymerization as described Weingartner *et al.* (2001); taxol (Sigma-Aldrich) was used at a concentration 5 µM. Inhibition of Cdk and Aurora kinase activity was done adding 100 µM roscovitine (a gift from Miroslav Strnad, Olomouc, Czech Republic) as described by Planchais *et al.* (1997) and Binarová *et al*. (1998) and 2 µM Aurora kinase inhibitor ZM447439 (Tocris Bioscience) as described by Ditchfield *et al.* (2003).

### Co-immunoprecipitation

Co-immunoprecipitations were performed using GFP-Trap A and RFP-Trap A (ChromoTek, Planegg-Martinsried, Germany) according to the manufacturer’s instructions using the modified protocol described in Petrovská *et al*. (2012). The extract from the *A. thaliana* cell culture expressing AtTPX2-GFP or co-expressing AtAurora1-RFP and AtTPX2-GFP (protein concentration 3–4mg ml^–1^) after centrifugation at 10,000 *g* for 10min were used directly or solubilized by 1% NP-40 for 1h at 4 °C. The extracts were supplemented with double concentration of inhibitors of proteases, with inhibitors of phosphatases and 50 µM MG132 (Sigma) and incubated with GFP-Trap or RFP-Trap beads for 1.5h at 4 °C. As a negative control, GFP immunoprecipitate from wild-type Ler *Arabidopsis* culture was used. The immunoprecipitated proteins were released by elution with glycine (pH 2.5). Proteins in the eluates were resolved by SDS-PAGE and analysed for importin, γ-tubulin, and AtAurora1 by immunoblotting with rabbit polyclonal anti-importin antibody 1:3000 (Secant Chemicals), affinity-purified rabbit polyclonal antibody AthTU 1:2,500 (Dryková *et al.*, 2003), anti-actin 1:1000 (Affinity BioReagents), anti-GFP and anti-RFP 1:2000 (Abcam and ChromoTek) antibodies, and anti-Ran antibody 1:200 (Transduction Laboratories). Secondary antibodies anti-rabbit and anti-mouse IgG HRP Conjugates (Promega or Amersham-GE Healthcare) were used; Super Signal West Pico Chemiluminiscent Substrate (Thermo Scientific) was used according to the manufacturer’s instructions.

### Immunofluorescence

*Arabidopsis thaliana* suspension cultures were fixed for 1h using 3.7% paraformaldehyde and processed for immunofluorescence as described in Binarová *et al*. (1993). Primary antibodies, anti-α-tubulin monoclonal antibody DM1A (Sigma) at a dilution of 1:500, monoclonal anti-γ-tubulin TU-32 (kindly provided by Pavel Dráber from IMG, Prague, Czech Republic) diluted 1:10, affinity purified rabbit polyclonal antibody AthTU 1:1000 (Dryková *et al.*, 2003), anti-GFP antibody (Abcam) at a dilution 1:1000, anti-actin (Affinity BioReagents) at a dilution 1:1000, anti-phospho-histone H3 (Ser10) antibody (Cell Signaling Technology) at a dilution 1:2000, monoclonal mouse anti-importin antibody (Secant Chemicals, Winchendon, MA) at a a dilution 1:2000, rabbit polyclonal anti-importin antibody (Secant Chemicals) at a dilution 1:3000, and anti-Ran antibody (Transduction Laboratories) at a dilution 1:200 were used with anti-mouse and anti-rabbit conjugated antibodies to FITC, DyLight 488, Cy3, DyLight 550, or Alexa Fluor 647 (Jackson ImmunoResearch Laboratories). DNA was stained with DAPI.

### In situ detection of fragmented DNA

The *In Situ* Cell Death Detection Kit (Roche) was used for the TUNEL (TdT-mediated dUTP nick-end labelling) test according to the manufacturer’s instructions. Besides the TUNEL test, the viability assay (on the basis of its penetration into non-viable cells) was determined by 10min incubation of cell suspension with 0.1% of Evans blue dye.

### Microscopy

Microscopy was performed using an IX81 motorized inverted research microscope CellR (Olympus) equipped with disk scanning unit and digital monochrome CCD camera CCD-ORCA/ER, and using an Olympus IX-81 FV-1000 confocal microscope. To avoid filter crosstalk, fluorescence was detected using HQ 480/40 exciter and HQ 510/560 emitter filter cubes for FITC and HQ 545/30 exciter and HQ 610/75 emitter filter cubes for Cy3 (both AHF Analysen Technique). Images were processed and analysed using CellR Software and Quick Photo Camera Software version 2.3 (Olympus). Images from confocal laser scanning microscopy were taken with PLAPO objective 100×/1.45 using the sequential multitrack mode to avoid bleed-through; excitation and emission wavelengths were 405 and 425–460nm for DAPI, 473 and 485–545nm for FITC or DyLight 488, 559 and 575–620nm for Cy3 or DyLight 550, and 635 and 655–755nm for Alexa Fluor 647. Green fluorescent protein was excited by 473nm and emission was detected from 485 to 545nm. Whenever needed, z-stacks were taken with 0.2 µm z-step. Images were analysed using FV10-ASW (Olympus); 3-D reconstruction and animation from z-stacks, and sectioning of gained 3D objects was performed using Imaris software (Bitplane) in the section and animation mode.

Figures were prepared using Adobe Photoshop 7.0. The quantitative colocalization analyses were performed using ImageJ software with JACoP (Just Another Co-localization Plug-in) plugin (Bolte and Cordeliéres, 2006) based on Pearson’s coefficient, overlap coefficient, and Manders’ coefficient (colocalization coefficient for channel M1, M2). Costes’ approach was expressed with a plot of the distribution of the Pearson’s coefficient of randomized images (curve) and of the green channel image (red line) and showed a probability of colocalization. Another development based on Pearson’s coefficient used for confirmation of a degree of colocalization was Van Steensel’s approach. Li’s approach were presented as a set of two graphs, each showing the normalized intensities (from 0 to 1) as a function of the product (A_i_ – a)(B_i_ – b) for each channel. Observed positive product (A_i_ – a)(B_i_ – b) and dot cloud concentrated on the right side of the *x* = 0 line (although adopting a C-shape) indicated high colocalization.

## Results

### *In silico* analyses suggested nuclear localization as well as nuclear function of AtTPX2.

**Fig. 1.**
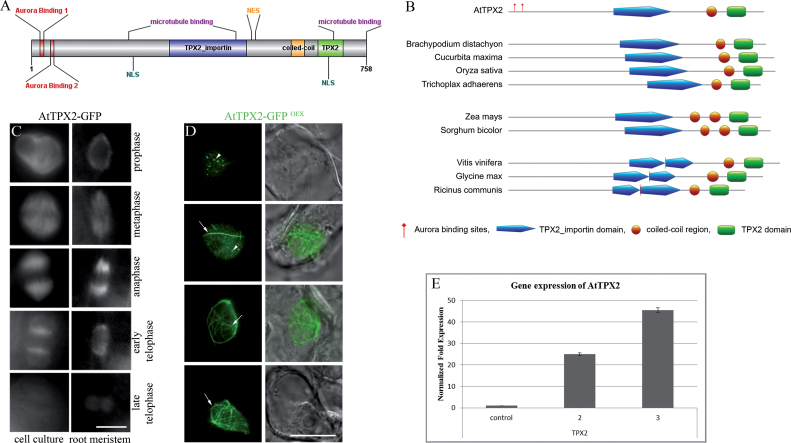
Localization of AtTPX2 protein in *Arabidopsis* cells overexpressing AtTPX2. (A) Domains, putative interaction sites, and binding motifs in the amino acid sequence of *Arabidopsis* TPX2 protein. For putative interaction sites, binding motifs as well as domain graph, ELM (Dinkel *et al.*, 2012), and SUMOsp 2.0 (Ren *et al.*, 2009) databases were used. Schematic drawing was prepared in DOG (Domain Graph; Xao and Xue, 2009). (B) Domain organization of *Arabidopsis* TPX2 protein and its plant homologues: AtTPX2–targeting protein for Xklp2–TPX2 (*Arabidopsis thaliana*, AGI no. At1g03780), *Brachypodium distachyon* uncharacterized protein LOC100842911 (acc. no. XP_003560168), *Cucurbita maxima* hypothetical protein (AEK84224), *Oryza sativa* hypothetical protein OsJ_24381 *Oryza sativa Japonica Group* (EEE67237), *Trichoplax adhaerens* hypothetical protein TRIADDRAFT_55817 (XP_002112111), *Zea mays* uncharacterized protein LOC100383389 (NP_001169515), *Sorghum bicolour* hypothetical protein SORBIDRAFT_02g034250 (XP_002462908), *Vitis vinifera* uncharacterized protein LOC100262517 (XP_002274918), *Glycine max* uncharacterized protein LOC100801192 (XP_003526269), *Ricinus communis* protein with unknown function (XP_002517880). Schematic drawing of proteins was prepared in MyDomains (Prosite, http://prosite.expasy.org/cgi-bin/prosite/mydomains/). (C) Localization of AtTPX2-GFP in dividing cell in cell culture with stable expression and in root meristematic zone of *A. thaliana*. AtTPX2-GFP was localized with mitotic microtubular arrays from prophase until early telophase. Bar, 10 µm. (D) Localization of AtTPX2-GFP in *Arabidopsis* cell suspension culture overexpressing AtTPX2-GFP. AtTPX2-GFP was predominantly localized with ‘dots’ or ‘seeds’ (arrowheads), later elongated into bundled fibres around (cage-like structures) and inside the nuclei (arrows). Frequency of overexpressing cells ranged between 10–40% depending on the transformation event, bar, 10 µm. (E) Relative expression of AtTPX2 in two representative samples of dividing *Arabidopsis* suspension cultures overexpressing AtTPX2 (samples 2, 3) showed a significant increase (25-fold, 45-fold, respectively) in transcript level compared to the control cells.

The AtTPX2 protein is composed of two domains: TPX2_importin (pfam: PF12214) and TPX2 domain (pfam: PF06886) (Fig. 1A) (Punta *et al.*, 2012). *Arabidopsis* TPX2 has two nuclear localization signals andm unlike other TPX2s, AtTPX2 has a signal for nuclear export (Vos *et al.*, 2008). AtTPX2 also possesses two domains that can mediate its localization to microtubules (Vos *et al.*, 2008; Petrovská *et al.*, 2012). The AtTPX2 protein also contains a short region (amino acids 588–619) that shows significant probability for coiled coil formation, as confirmed using several algorithms: Coils (Lupas *et al.*, 1991), Paircoil (Berger *et al.*, 1995), MultiCoil (Wolf *et al.*, 1997), and ELM (Dinkel *et al.*, 2012). The coiled coil region was found in plant proteins that contain TPX2_importin and TPX2 motifs across various plant species (Fig. 1B); most of them belonged to as-yet uncharacterized or hypothetical plant proteins.

Deeper analysis of *Arabidopsis* TPX2 amino acid sequence (Dinkel *et al.*, 2012) revealed the following: the presence of an HP1 ligand (interacts with chromoshadow domain of heterochromatin-binding protein 1, amino acids 95–99), KEN box (148–152, 275–279, and 667–671), D box (327–645), three cyclin recognition sites (305–309, 332–336, 516–519), several FHA phosphopeptide ligands (predominant in nuclear proteins that are involved in cell cycle checkpoint, DNA repair and transcriptional regulation), mitotic spindle checkpoint protein MAD2 binding motif (361–369), mitogen-activated protein kinase, docking motifs (41–47, 492–502, 538–549, 723–731), sumoylation sites (68–71, 208–211, 529–532, 575–578), and several phosphorylation sites (i.e. PIKK, glycogen synthase kinase 3, PKA; Supplementary Table S1, available at *JXB* online). Localization of proteins with these motifs or interaction sites is typically nuclear. Plant TPX2 is localized in nuclei (Vos *et al.*, 2008; Petrovská *et al.*, 2012). In addition, using Nuc-PLoc (Hong-Bin and Kuo-Chen, 2007) and Subnuclear Compartments Prediction System 2.0 (Lei and Dai, 2005, 2006), the subnuclear localization of *Arabidopsis* TPX2 protein was predicted to be nuclear speckle and nuclear lamina, respectively.

### Fibres heavily decorated with TPX2 are formed in the vicinity of the nuclear envelope and in the nuclei of cells overexpressing AtTPX2

Cultured cells and seedlings of *A. thaliana* were transformed with plasmids containing full-length C- and N- terminal AtTPX2 protein fusions with GFP, under the control of the cauliflower mosaic virus (CaMV) 35S constitutive promoter. After selection, stable cell lines were derived (Petrovská *et al.*, 2012). Both C- and N- terminal AtTPX2-GFP fusion proteins showed similar localizations. The AtTPX2-GFP was observed in nuclei, the perinuclear region and, in a cell cycle-specific manner, with mitotic microtubular arrays. The localization of AtTPX2-GFP fusion protein was similar for dividing cultured cells and cells of *Arabidopsis* seedlings (Fig. 1C). The AtTPX2-GFP signal was present with perinuclear microtubules in preprophase, with kinetochore microtubular fibres in metaphase and anaphase, and with early phragmoplast in cytokinesis. It was previously found that the cell cycle-specific microtubular localization of AtTPX2-GFP requires Aurora binding (Petrovská *et al.*, 2012). TPX2 knockout in *Arabidopsis* is lethal and only heterozygous plants could be obtained for the T-DNA inserts (Vos *et al.*, 2008; data not shown). Therefore, the current study was not able to test functionality of the AtTPX2-GFP protein under the most stringent conditions by complementation of null mutants. However, the localization data and interaction of AtTPX2-GFP with Aurora kinase (Petrovská *et al.*, 2012) suggest that the AtTPX2-GFP fusion protein is functional.

As early as 48h after transformation, expressed fusion protein showed diffused distribution in cytoplasm with accumulation in nuclei; a later AtTPX2 signal was predominantly associated with dots and patches in nuclei and as fibrillar structures located inside the nuclei and in the perinuclear area (Fig. 1D). The effects of overproduction of AtTPX2 N- and C-terminal GFP fusion proteins were similar, suggesting that the GFP moiety did not interfere with the function of AtTPX2 in fibre formation. Overexpression of AtTPX2-GFP was confirmed by real-time qPCR (Fig. 1E). Microscopic analysis of AtTPX2-overexpressing cells showed that the dots of AtTPX2-GFP in interphase nuclei (Fig. 1D, arrowheads) were formed within a period of 3 d from transformation. AtTPX2-GFP dots and patches were gradually built into thick fibrillar structures (Fig. 1D, arrows). The AtTPX2-GFP signal was attached to filamentous structures reminiscent of microtubules that were arranged into cage-like structures surrounding the nuclei (Fig. 1D, arrows).

**Fig. 2.**
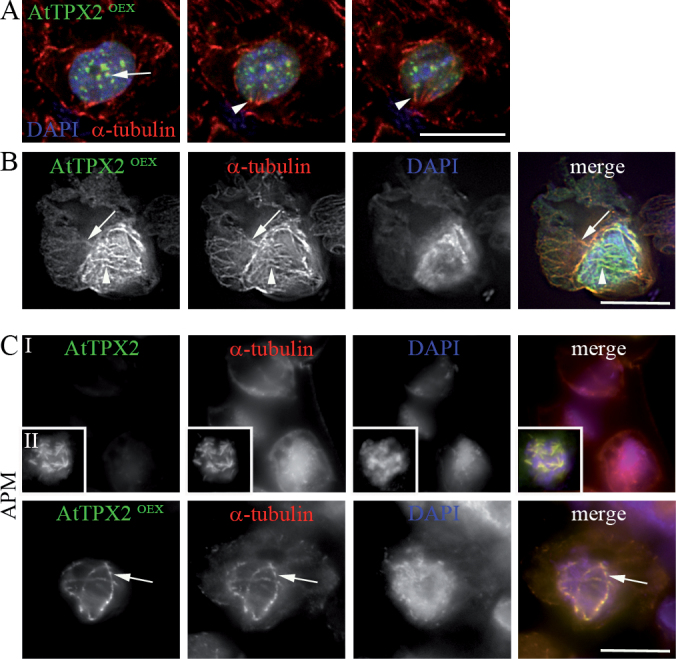
AtTPX2 localization with ectopic perinuclear and nuclear microtubules in *Arabidopsis* cells overexpressing AtTPX2-GFP. (A) AtTPX2-GFP foci localized with α-tubulin in the nucleus (arrow) and AtTPX2-GFP partially loaded on α-tubulin fibres (arrowheads) in cells with foci of overexpressed AtTPX2-GFP. Serial sections (0.4 µm z-steps) of the cell immunostained by anti-GFP antibody (green) and anti-α-tubulin antibody (red) with chromatin stained by DAPI (blue) are shown. (B) α-Tubulin signal with thinner AtTPX2-GFP-decorated fibres (arrow) was stronger compared to the signal with thick bundles (arrowhead); 99% of analysed cells showed the corresponding pattern (194 analysed cells). (C) While microtubules were depolymerized, with exception of kinetochore stubs, in cells expressing AtTPX2-GPF after the treatment with 5 µM APM for 2.5h, microtubular arrays in cell overexpressing AtTPX2-GFP (AtTPX2^OEX^) were stable after the same treatment. Bars, 10 µm.

To prove whether AtTPX2-decorated fibres in AtTPX2-overexpressing cells represent cytoskeletal polymers, this study performed a series of double immunofluorescence experiments. Fibres were positive for α-tubulin (Fig. 2A, B), but they were not recognized by anti-actin antibody (Supplementary Fig. S1). The signal for α-tubulin localized with the AtTPX2 foci in nuclei (Fig. 2A, arrow), and with forming fibres (Fig. 2A, arrowheads). In AtTPX2-overexpressing cells containing more prominent fibrillar arrays, α-tubulin was associated with thinner AtTPX2-positive fibres along their entire length (Fig. 2B, arrow). Thicker bundles, heavily labelled with AtTPX2-GFP, showed a weaker signal for α-tubulin (Fig. 2A, arrowheads), probably due to the lower accessibility of the epitope to the anti-α-tubulin antibody.

Treatment of cells overexpressing AtTPX2 with the microtubule-depolymerizing drug APM showed that the nuclear and perinuclear microtubular bundles were resistant to drug-induced depolymerization. As shown in Fig. 2C, the microtubular arrays persisted in 98% of 5 µM APM- treated cells (*n* = 112), showing overexpression of AtTPX2-GFP. Microtubules were largely depolymerized by the same dose of APM in cells with stable expression of AtTPX2-GFP (Fig. 2C, inset I) and only remnants of kinetochore microtubules decorated with AtTPX2-GFP were observed (Fig. 2C, inset II). Taxol treatment did not result in further bundling or stabilization of ectopic microtubules in AtTPX2-GFP-overexpressing cells (Supplementary Fig. S2).

**Fig. 3.**
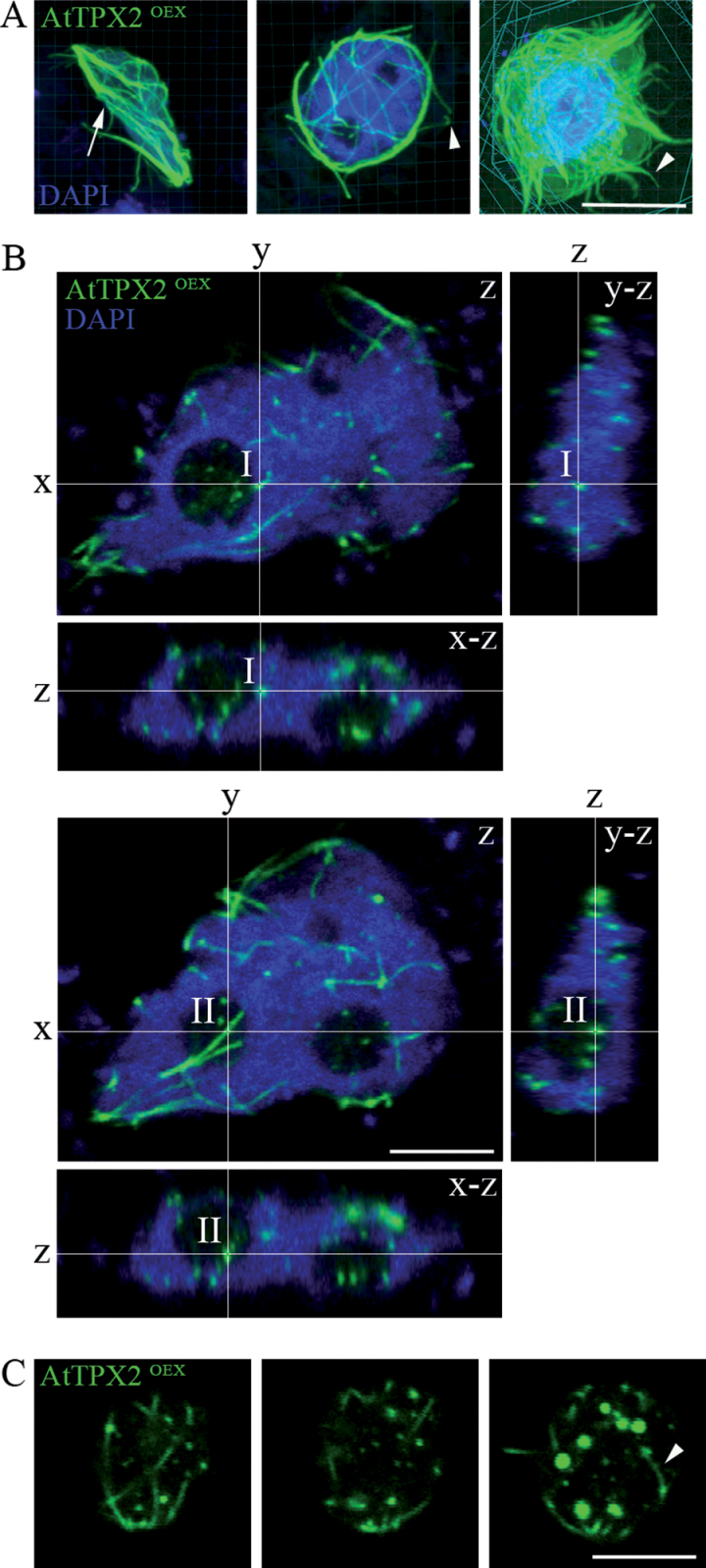
Three-dimensional reconstruction and analysis of microtubular arrays in *Arabidopsis* cells overexpressing AtTPX2-GFP. (A) Representative images from 3D reconstruction of cells with AtTPX2-decorated fibres around and inside nuclei; Microtubular fibres in the perinuclear area are twisted (arrow), the microtubular fibres anchored nuclei to cell periphery (arrowheads); bar, 10 µm. (B) Two Imaris sections of the nucleus with AtTPX2-decorated fibres; AtTPX2-decorated fibres were present inside the nucleus (cross I) and the nucleolus (cross II) overexpressing AtTPX2; main panel *z* shows a single z-stack of the nucleus; right panel *y*-*z* shows cross-section by *y* plane perpendicular to *z* plane in the main panel; lower panel *x-z* shows cross-section by *x* plane perpendicular to *z* plane in the main panel; bar, 5 µm. (C) Serial z-stacks of nucleus of cell overexpressing AtTPX2-GFP analysed *in vivo*. Sections from nuclear surface to the centre (left to right) showed perinuclear fibres and intranuclear foci and fibres decorated by AtTPX2 (arrowhead); bar, 5 µm.

Serial sections of nuclei and 3-D reconstructions showed a network of microtubules decorated with AtTPX2 inside the nuclei and in the area adjacent to the nuclei (Fig. 3A). Thick intertwined bundles of microtubules were present around the nuclei (Fig. 3A, arrow) and anchored to the nucleus at the cell periphery (Fig. 3A, arrowheads). More detailed analyses using Imaris sectioning of nuclei of AtTPX2-GFP-overexpressing cells confirmed the formation of fibrillar structures inside the nuclei, in the vicinity of chromatin (Fig. 3B, cross I) and also inside the nucleoli (Fig. 3B, cross II). To prove nuclear location of ectopic arrays, cells overexpressing AtTPX2-GFP *in vivo* were analysed. As shown in Fig. 3C, fibres decorated with AtTPX2-GFP were observed, together with intranuclear foci of overexpressed TPX2.

These data showed that overexpressed AtTPX2-GFP protein was initially present in nuclear foci and patches, and later, fibrillar microtubular arrays were formed in nuclei and the perinuclear area. The ectopic microtubular fibres, heavily decorated with AtTPX2-GFP, were resistant to microtubular drugs.

### Interaction of importin with AtTPX2-GFP suggests involvement of the Ran cycle in TPX2-mediated formation of microtubular arrays.

**Fig. 4.**
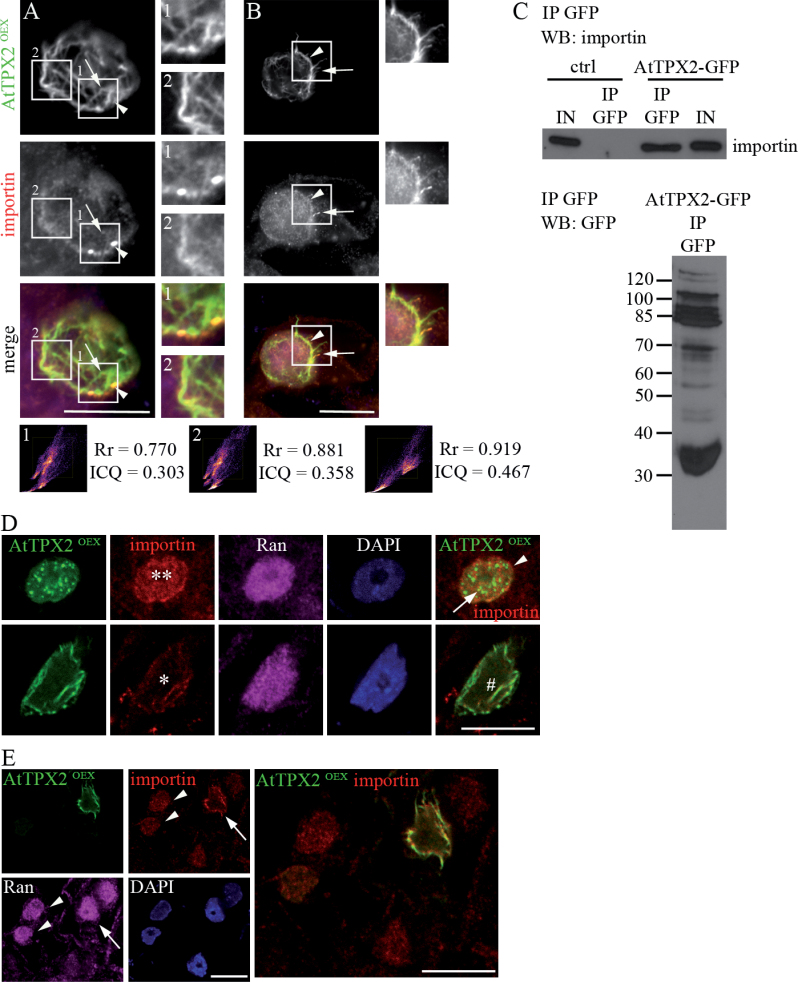
Immunolocalization of importin and Ran in AtTPX2-GFP-overproducing cells. (A, B) AtTPX2 and importin colocalized in the vicinity of the nuclei (B, arrowhead), with the AtTPX2-decorated microtubular fibres (A, B, arrows), and in intranuclear foci (A, arrowhead). Colocalization was analysed by ImageJ software with JACoP plugin (Bolte and Cordeliéres, 2006). The similar labelling pattern was observed in 97% of analysed overexpressing cells (*n* = 112). (C) Importin was copurified with AtTPX2-GFP by GFP trap (AtTPX2-GFP IP GFP) from extracts of *Arabidopsis* cells. GFP immunoprecipitate from wild-type Ler *Arabidopsis* culture was used as a negative control (ctrl IP GFP). Immunoblotting of GFP immunoprecipitate from AtTPX2-GFP expressing cell culture with anti-GFP antibody showed several bands corresponding to the full-length molecule of AtTPX2-GFP above 100kDa and several degradation products. (D, E) Representative images of immunofluorescence labelling of the cells overexpressing AtTPX2-GFP (green), immunostained with anti-importin (red), with anti-Ran (magenta); chromatin stained by DAPI (blue). (D) Importin signal was nuclear (two asterisks), localized around nuclei (arrowhead) and colocalized with some of the AtTPX2 foci (arrow) in overexpressing cells with AtTPX2-GFP foci and patches. Nuclear signal for importin was reduced (asterisk) in cells where AtTPX2-GFP fibres were formed and it localized with the fibres around nuclei and in the nuclei (hash mark). (E) Signal for Ran in the nuclei of cells with AtTPX2 perinuclear fibres was not above the level of signal found in untransformed cells (arrowheads). Importin nuclear signal declined in the cells with AtTPX2-GFP fibres (arrow) while a portion of signal for importin localized with AtTPX2 perinuclear fibres. Bars, 10 µm.

Importin binds TPX2 protein and imports it into the nucleus, and RanGTPase sequesters the TPX2 nuclear pool before breakdown of the nuclear envelope (Gruss *et al.*, 2001). Binding of animal importin to recombinantly expressed plant TPX2 protein in a RanGTPase-dependent manner was shown *in vitro* by Vos *et al.* (2008). To determine whether the RanGTPase pathway is involved in the process of AtTPX2-mediated formation of microtubular arrays, the current work analysed the immunolocalization of importin and Ran in AtTPX2-GFP-overproducing cells (Fig. 4 and Supplementary Fig. S3). The signal for importin was observed in nuclei, associated with the nuclear envelope, and with intranuclear and perinuclear microtubular fibres (Fig. 3). Quantitative co-localization analyses of AtTPX2 and importin were performed using the ImageJ plugin, JACoP (Bolte and Cordeliéres, 2006). The analyses showed high degrees of co-localization of AtTPX2-GFP, with patchy patterns of importin on perinuclear fibres (Fig. 4A, arrowhead), and with microtubular fibres extending from the perinuclear area to the periphery (Fig. 4B, arrow). All coefficients correctly reported a strong overlap between the two channels (Supplementary Fig. S5).

Previous experiments used GFP co-immunoprecipitation to show an interaction of AtAurora1-RFP with AtTPX2-GFP (Petrovská *et al.*, 2012). The current work performed GFP co-immunoprecipitation to provide evidence for an interaction between AtTPX2-GFP and importin. As shown in Fig. 4C, importin was co-purified with AtTPX2-GFP from low-speed supernatants. Negative controls as well as probing of purified TPX2 complexes with the relevant anti-actin antibody confirmed that interaction of importin with AtTPX2 was specific (Fig. 4C, Supplementary Fig. S4). It is known that AtTPX2 protein is highly unstable in plant cell extracts or under conditions of electrophoretic protein separation, and this makes detection by Western blotting difficult (Vos *et al.*, 2008; Petrovská *et al.*, 2012). In agreement with data shown by Vos *et al.* (2008) and Petrovská *et al*. (2012), several bands for the AtTPX2-GFP protein and its degradation products were detected with anti-GFP antibody in a sample of immunopurified AtTPX2-GFP (Fig. 4C).

Next, Ran protein was immunolocalized in AtTPX2-overexpressing cells. The signal for Ran was stronger in nuclei with AtTPX2-GFP nuclear dots and patches, where 83% of nuclei (*n* = 132) showed higher levels of the Ran signal compared to the untransformed controls (Supplementary Fig. S3). On the other hand, only 23% of nuclei (*n* = 94) showed any signal above that of control untransformed nuclei in cells with nuclear/perinuclear microtubular arrays (Fig. 4E). This finding suggests that the Ran nuclear signal was weakening during microtubular array assembly. Multiple labelling showed that, similarly to Ran, the importin signal was enriched in nuclei of overexpressing cells where it co-localized with some of the AtTPX2-GFP foci and accumulated around NE (Fig. 4D, arrow and arrowhead). In cells with assembled fibres, diminution of the nuclear signal for importin was even more pronounced compared to that observed for Ran (Fig. 4D, E). A smaller proportion of the importin signal was observed associated with the AtTPX2-GFP-decorated microtubular arrays.

**Fig. 5.**
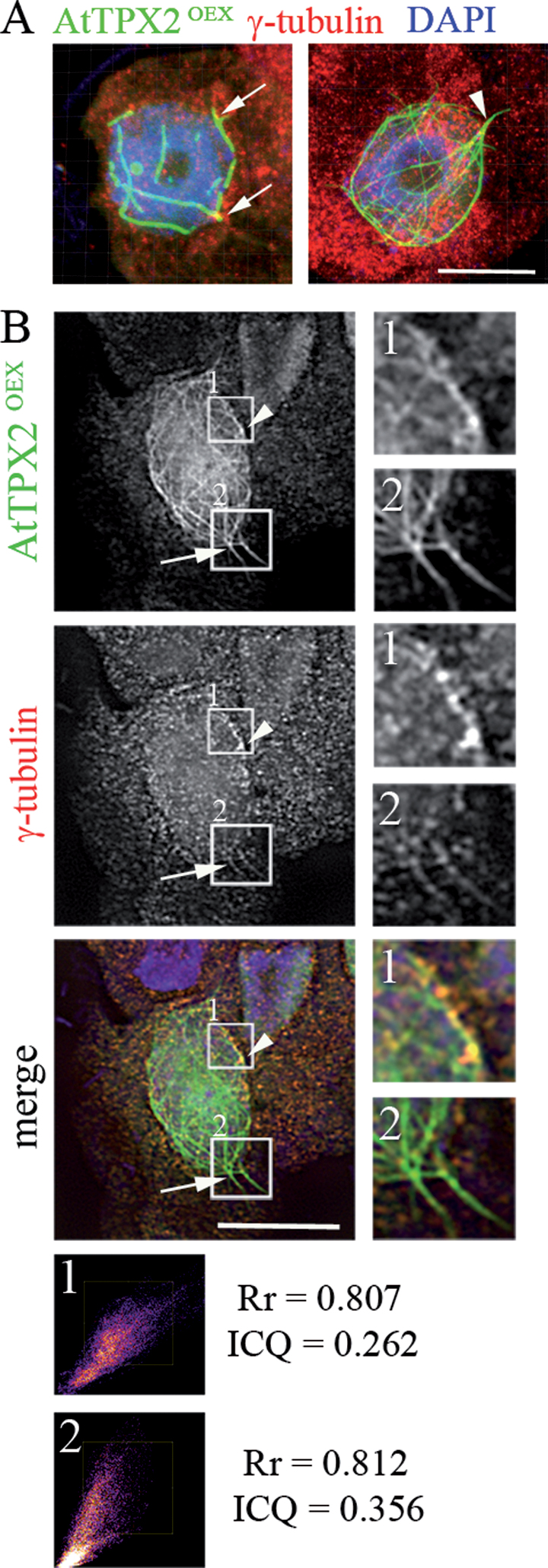
Colocalization of AtTPX2 and γ-tubulin in *Arabidopsis* AtTPX2-GFP-overproducing cells. (A, B) γ-Tubulin localized together with AtTPX-GFP. (A) 3D analysis of cells overexpressing AtTPX2-GFP stained with anti-γ-tubulin antibody (red), DAPI (blue) obtained by laser scanning microscopy and 3D-reconstructed (Imaris, Bitplane). Still images from 3D reconstruction animation show γ-tubulin localized with AtTPX2-GFP-decorated fibres in vicinity of nuclear envelope (A, arrows), with intranuclear microtubules, and in patchy pattern on microtubular bundles extending from perinuclear area (arrowhead). (B) γ-Tubulin colocalized with AtTPX2 on microtubular fibres extending from perinuclear area to the cytoplasm and membranes (arrow) and on the nuclear envelope (arrowhead). Colocalization was analysed by ImageJ software with JACoP plugin (Bolte and Cordeliéres, 2006). The similar labelling pattern was observed in 93% of analysed overexpressing cells (*n* = 128). Bars, 10 µm.

Plant γ-tubulin is associated with microtubular arrays, around the nuclear envelope, and in nuclei (Dryková *et al.*, 2003). Petrovská *et al*. (2012) showed that γ-tubulin is localized with AtTPX2 and AtAurora1 on mitotic microtubular arrays. Therefore, this work analysed the localization of γ-tubulin in cells overexpressing AtTPX2-GFP. Signals for γ-tubulin were observed in a patchy pattern with microtubular bundles formed in the vicinity of the nuclei and extending to the cell periphery (Fig. 5A). Quantitative co-localization analyses of γ-tubulin and AtTPX2, using the ImageJ plugin JACoP, showed co-localization of γ-tubulin with AtTPX2-GFP-decorated microtubular arrays (Fig. 5B1, 2). However, the association of γ-tubulin with immunopurified AtTPX2-GFP could not be demonstrated.

Together, the immunolocalization and immunopurification data suggest a function for importin with plant AtTPX2. The accumulation of importin and Ran in the nuclei of overexpressing cells indicates a nuclear import of overexpressed AtTPX2-GFP. Diminution of the nuclear signal for importin, as observed in cells with microtubular arrays, indicates that microtubule formation was triggered by sequestered AtTPX2, possibly Ran cycle-dependent. The presence of an importin signal with AtTPX2 on ectopic microtubular fibres suggests that an excess of the overexpressed AtTPX2 may still be bound to importin.

### Formation of AtTPX2-decorated microtubular fibres was neither dependent on association of Aurora kinase with TPX2 nor on mitotic status of the chromatin.

**Fig. 6.**
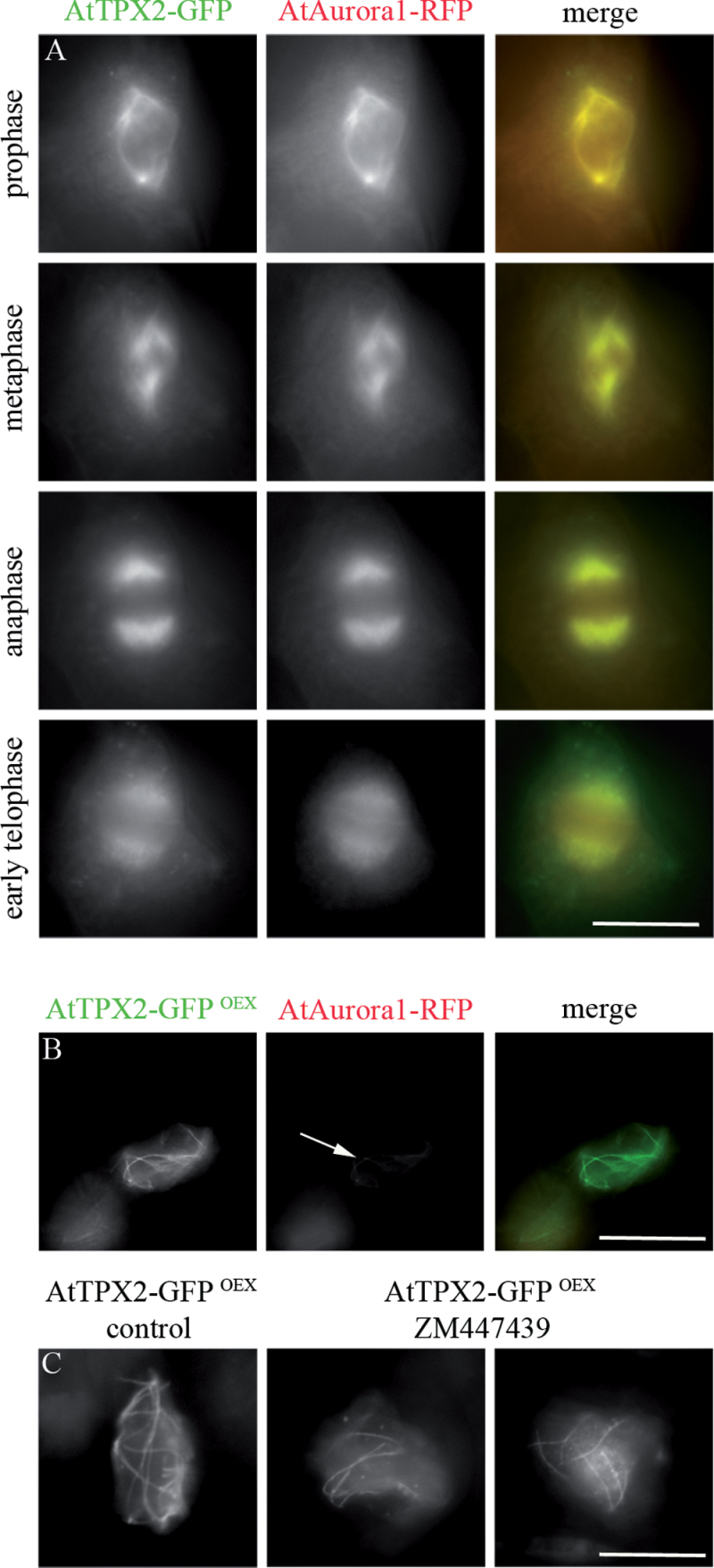
Localization of AtTPX2 and AtAurora1 in control and ZM447439-treated cells overproducing AtTPX2. (A) Localization of AtTPX2-GFP and AtAurora1-RFP from the prophase until the early telophase in *Arabidopsis* cells. (B) Weak signal of AtAurora1 with AtTPX2 (arrow) in the nuclei of *Arabidopsis*. (C) AtTPX2 fibres in TPX2-overproducing cells were resistant to Aurora kinase inhibitor ZM447439 (100% of analysed cells showed resistance to ZM447439, *n* = 96). Bars, 10 µm.

Previous data showed that AtAurora1 binds to AtTPX2 and that binding is required for cell cycle-specific localization of TPX2 on mitotic microtubular arrays (Petrovská *et al.*, 2012). In cells with stable expression of AtTPX2-GFP and AtAurora1-RFP, both proteins colocalized with microtubular arrays from preprophase to early telophase (Fig. 6A) and were co-purified (Supplementary Fig. S6). However, only a weak AtAurora1 signal was found on bundled AtTPX2-decorated microtubules in overexpressing cells (Fig. 6B). To determine whether binding of Aurora kinase was required for TPX2-mediated formation of ectopic microtubular arrays, this work overexpressed a truncated version of AtTPX2 that lacked two conserved Aurora kinase binding sites (ΔN-AtTPX2; Petrovská *et al.*, 2012). Microtubular arrays were formed in the vicinity of and in the nuclei, and were associated with ΔN-AtTPX2 (Supplementary Fig. S7). This work analysed the effect of the Aurora kinase inhibitor, ZM447439 (Ditchfield *et al.*, 2003) that affected microtubular mitotic arrays in wild-type *Arabidopsis* cells (Supplementary Fig. S8). Contrary to the wild-type cells, ZM447439 treatment had no visible effect on the formation, stability, and arrangement of nuclear and perinuclear microtubular bundles in cells overexpressing AtTPX2 (Fig. 6C). These data suggest that binding of Aurora kinase with AtTPX2, which is required for localization of TPX2 on mitotic microtubules, is dispensable for the formation and organization of the ectopic peri/intranuclear microtubules in AtTPX2-overexpressing cells.

**Fig. 7.**
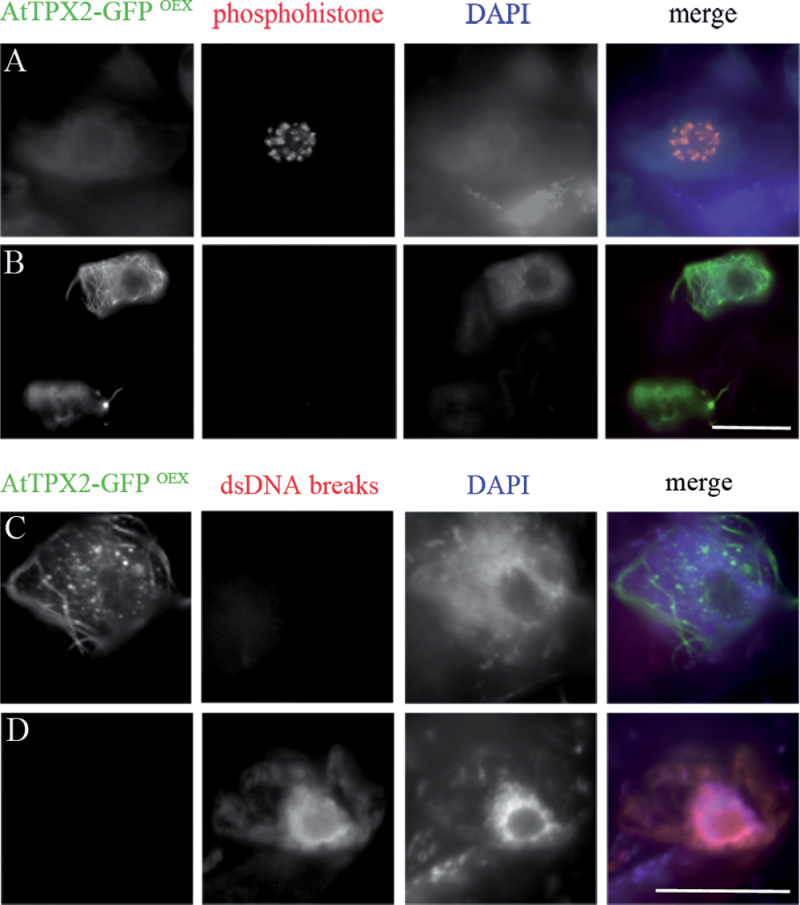
Formation of AtTPX2 fibrillar structures was not dependent on mitotic status of the chromatin or connected with programmed cell death. (A, B) Immunolabelling of AtTPX2 (green) and phosphohistone (red) in prophase (A) and interphase (B) cells overexpressing AtTPX2. AtTPX2-decorated ectopic microtubules were not formed in nuclei with mitotic chromatin. (C, D) Ectopic AtTPX2-decorated microtubular arrays were not observed in cells entering programmed cell death that were positive with TUNEL labelling (red). Bars, 10 µm.

To understand whether the formation of ectopic microtubules was cell cycle specific, anti-phosphohistone H3 (Ser10) antibody was used to monitor the mitotic status of the chromatin, from pre-prophase to metaphase (Fig. 7A). Cells (*n* > 100) with AtTPX2-decorated fibres did not show phosphohistone staining (Fig. 7B). Further proof that fibres were not formed in preparation for mitosis was provided by DAPI staining that showed diffuse interphase chromatin but not pre-mitotic condensed chromatin in cells with the TPX2-decorated microtubular arrays. The formation and arrangement of ectopic microtubular bundles was not affected by treatment with the mitotic kinase inhibitor roscovitine (Supplementary Fig. S9), which previously showed a severe effect on microtubular arrays (Binarová *et al.*, 1998). These findings suggest that ectopic nuclear and perinuclear microtubules were not formed specifically during the transition from interphase to mitosis.

### AtTPX2-stabilized microtubules are not found in cells with programmed cell death.

A type of AtTPX2-stabilized microtubular array similar to that observed in these experiments is formed in mammalian cells where it functions during apoptosis (Moss *et al.*, 2009). The current examined whether the TPX2-induced microtubules were involved in apoptosis in *Arabidopsis*. *In situ* detection of dsDNA breaks (TUNEL test) was used. The proportion of cells with TUNEL-stained nuclei was 8% in both wild-type (*n* = 234) and AtTPX2-overexpressing cells (*n* = 217). Neither AtTPX2 dots nor ectopic perinuclear or nuclear microtubules were observed in cells with TUNEL-detectable dsDNA breaks (Fig. 7C, D). To provide further evidence of programmed cell death, cell viability was analysed using Evans blue staining, which does not discriminate between apoptosis and necrosis (Danon *et al.*, 2000); however, changes in the plasma membrane recognized by Evans blue might be an early indicator of cells undergoing DNA fragmentation prior to TUNEL-detectable DNA breakage. Overexpressing cells with AtTPX2-GFP-labelled fibres were not stained with Evans blue (Supplementary Fig. S10). The formation of AtTPX2-decorated ectopic microtubules was therefore not associated with programmed cell death.

## Discussion

Previous studies showed that AtTPX2 was associated with spindle microtubules and suggested a microtubular function (Vos *et al.* 2008; Petrovská *et al.*, 2012). Analysis of cells overexpressing AtTPX2 protein allowed the identification of stable microtubular arrays ectopically nucleated in the nuclei and their periphery. Signals for overexpressed AtTPX2-GFP were initially observed in nuclear dots and patches, from which microtubules could grow and organize. Intranuclear and perinuclear AtTPX2-decorated fibrillar arrays were assembled later and formed a cage enveloping the nucleus and extending to the cell periphery.

Ran GTPase influences microtubule dynamics in mitosis by releasing spindle assembly factors from importins in the vicinity of chromatin (Joseph, 2006). There are several lines of evidence suggesting that TPX2-mediated microtubule formation observed under conditions of overproduction of AtTPX-GFP was triggered by a Ran nucleo-cytoplasmic gradient: (i) colocalization and co-immunopurification of importin with AtTPX2; (ii) accumulation of Ran and importin in the nuclei with overexpressed AtTPX2-GFP; and (iii) reduction of nuclear signals for Ran and importin that occurred simultaneously with assembly of nuclear/perinuclear microtubular arrays. Dynamic release of TPX2 from importin by Ran is active in organization of microtubules during mitosis in animals (Gruss *et al.*, 2001; Kufer *et al.*, 2002). The current data are in agreement with those on Ran GTP-dependent interaction of animal importin with recombinantly expressed plant TPX2 *in vitro* and on nucleo-cytoplasmic shuttling of the AtTPX2-GFP (Vos *et al.*, 2008). In addition to prominent nuclear and perinuclear signals for importin, the current work immunolocalized importin with TPX2-decorated ectopic microtubular arrays. While the ability of TPX2 to nucleate microtubules is abolished by binding of importin, the binding does not prevent TPX2 interaction with tubulin or with microtubules (Schatz *et al.*, 2003). The current work suggests that the importin associated with TPX2-decorated microtubules might represent the proportion of overexpressed AtTPX2 protein that was not sequestered from importin.

These data contribute to the understanding of TPX2-mediated microtubule formation in plants and suggest that the process is regulated by the Ran cycle. Most components of the Ran cycle were identified in plants and the regulatory role of Ran in cell division is, at least partially, conserved (Pay *et al.*, 2002; Jeong *et al.*, 2005). Direct visualization of the RanGTP gradient in living cells (Kalab *et al.*, 2006) has not been performed in plants. Ran FRET sensors, together with *in vivo* analysis of importin/TPX2 shuttling, are required to understand the function of the Ran cycle in microtubule formation and cell division in plants.

Microtubular nucleation in acentrosomal plants occurs from dispersed γ-tubulin-positive sites located on the nuclear envelope, in nuclei, and on pre-existing microtubules (Binarová *et al.*, 2000, 2006; Murata *et al.*, 2005; Nakamura *et al.*, 2010). The current data indicate that rearrangement from the ‘seeds’ through the bundled fibres might be caused by co-assembly of AtTPX2-GFP with endogenous microtubule-nucleating units, comprising γ-tubulin and TPX2 protein. A possible role of AtTPX2 with plant microtubules was indicated by previous data on colocalization of γ-tubulin with active AtAurora1/AtTPX2 on mitotic microtubular arrays (Petrovská *et al.*, 2012). Recently, it has been shown *in vitro* in *Xenopus* extracts that nucleation of branched microtubules from pre-existing microtubules requires γ-tubulin, TPX2, and augmins (Petry *et al.*, 2013). Colocalization of γ-tubulin with overexpressed AtTPX2 observed in the current work, indicating that a similar mechanism functions in TPX2-mediated formation of microtubular arrays. However, no association of γ-tubulin with immunopurified AtTPX2-GFP could be demonstrated. The low stability of plant TPX2 in extracts (Vos *et al.*, 2008; Petrovská *et al.*, 2012) might also influence the efficiency of immunopurification protocols. Furthermore, as was shown by co-immunoprecipitation by Petry *et al.* (2013), γ-tubulin is only a minor interactor with TPX2 protein. Further experiments are needed to demonstrate γ-tubulin interaction with TPX2 and to show how TPX2 cooperates with nucleation machinery in plant cells.

Similarly to animal homologues, plant TPX2 belongs to the group of cell cycle-regulated molecules that accumulate in the nuclei at the G2 phase of the cell cycle and are degraded at anaphase-telophase (Vos *et al.*, 2008). The current work found that AtTPX2-mediated microtubule formation did not require a mitotic status of chromatin, and thus is not reminiscence of Ran GTPase-dependent chromatin-mediated spindle formation in *Xenopus* extracts (Heald *et al.*, 1996). Furthermore, formation of TPX2-mediated fibres was not dependent on the binding of Aurora kinase to AtTPX2 and correspondingly was not sensitive to Aurora kinase inhibition. These data suggest that the tight tuning that depends on the cell cycle and Aurora kinase signalling to TPX2 is missing during formation of the ectopic microtubular arrays and is overdominated by AtTPX2 in microtubule nucleation and stabilization.

The TPX2-mediated microtubule nucleation pathway, regulated by Ran, is responsible for the assembly of specific acentrosomal microtubular arrays in apoptotic HeLa cells (Moss *et al.*, 2009). The TPX2-stabilized microtubular arrays functioning in apoptosis strongly resemble the stable AtTPX2-mediated microtubular arrays that was observed in plants. However, the arrays were not found in cells undergoing programmed cell death (TUNEL-positive cells) or in Evans blue-stained cells. As shown by Moss *et al.* (2009), TPX2-dependent microtubular arrays function in fragmentation of the HeLa cells nuclei during late apoptosis. While the molecular mechanisms of early apoptosis in plant and animal cells are more similar than was previously thought, the execution phase of the process differs. Electron microscopy of root initials in *Arabidopsis* showed that death of the stem cells did not show apoptotic features such as peripheral chromatin condensation and nuclear fragmentation (Fulcher and Sablowski, 2009). The TPX2-mediated formation of microtubular arrays is exploited by animal and plant cells alike, but the function of these acentrosomal microtubular arrays may reflect specific needs of the organism and cell type.

The *in silico* analysis of AtTPX2 showed the presence of a coiled-coil domain, specific motifs, interaction sites, and predicted subnuclear localization. In metazoans, coiled-coil proteins group as various cytoskeletal networks comprising intermediate filament proteins, actin-binding proteins, and microtubule-associated proteins (Burkhard *et al.*, 2001; Korolev *et al.*, 2005). Albeit several proteins closely related to the intermediate filament protein were identified *in silico* (Gardiner *et al.*, 2011), knowledge of plant coiled-coil proteins is limited. The current work found that overproduced AtTPX2 accumulated in interphase nuclei and observed TPX2-dependent fibrillar arrays interconnecting nuclei with the nuclear periphery. A role for RanGTP in forming a lamin B spindle matrix has been reported (Tsai *et al.*, 2006) and TPX2 was shown to be required for post-mitotic nuclear envelope assembly (O’Brien and Wiese, 2006). The current study can only speculate as to whether AtTPX2 connects as-yet undefined plant cell lamina with the cytoskeleton and plays a role during interphase as a component of a plant alternative to the LINC (linker of nucleoskeleton and cytoskeleton) complex (Crisp *et al.*, 2006; Tzur *et al.*, 2006).

In summary, the overexpression of AtTPX2-GFP resulted in the formation of chromatin- and nuclei-associated microtubular arrays. The assembly of TPX2-decorated fibres was dependent on neither the mitotic status of chromatin nor the binding of Aurora kinase. The arrays were not specific to apoptotic cells. This study suggests that AtTPX2 overexpression amplified an ability of the nuclear envelope and chromatin to promote microtubule nucleation that is typical for acentrosomal plant cells. Furthermore, these findings indicate an involvement of the Ran pathway in modulation of the process.

## Supplementary material

Supplementary data are available at *JXB* online.

Supplementary Table S1. *In silico* analyses of AtTPX2 protein.

Supplementary Fig. S1. Immunolocalization of AtTPX2 and actin in cell cultures of *Arabidopsis thaliana.*

Supplementary Fig. S2. AtTPX2-decorated fibres are resistant to taxol in cell cultures of *Arabidopsis thaliana.*

Supplementary Fig. S3. Immunofluorescence localization of Ran in AtTPX2-GFP-overproducing cells.

Supplementary Fig. S4. Importin copurified with AtTPX2-GFP from *Arabidopsis* cultured cells.

Supplementary Fig. S5. Colocalization analyses of AtTPX2 and importin in *Arabidopsis* cultured cells.

Supplementary Fig. S6. AtAurora1-RFP copurifies with AtTPX2-GFP from *Arabidopsis* cultured cells.

Supplementary Fig. S7. Overexpression of AtTPX2 and ΔN-AtTPX2 in *Arabidopsis* nuclei.

Supplementary Fig. S8. Treatment of mitotic microtubules with Aurora kinase inhibitor ZM447439 in cell cultures of *Arabidopsis thaliana.*

Supplementary Fig. S9. Ectopic nuclear microtubular bundles were not affected by roscovitine treatment in cell cultures of *Arabidopsis thaliana.*

Supplementary Fig. S10. Evans blue viability test in *Arabidopsis* cell cultures with overproduced AtTPX2.

Supplementary Data
