# Supplementary material for: Overexpressed TPX2 causes ectopic formation of microtubular arrays in the nuclei of acentrosomal plant cells
Source: J Exp Bot. 2013 Sep 4;64(14):4575–87. doi: 10.1093/jxb/ert271 (PMC3808333; doi:10.1093/jxb/ert271)
Supplement: Supplementary Data [file supp_64_14_4575__index.html]

Overexpressed TPX2 causes ectopic formation of microtubular arrays in the nuclei of acentrosomal plant cells — Supplementary Data 

# Overexpressed TPX2 causes ectopic formation of microtubular arrays in the nuclei of acentrosomal plant cells

## Supplementary Data

Data files

**Files in this Data Supplement:**

- Supplementary Data - Supplementary Data
