# Supplementary material for: Overexpressed TPX2 causes ectopic formation of microtubular arrays in the nuclei of acentrosomal plant cells
Source: J Exp Bot. 2013 Sep 4;64(14):4575–87. doi: 10.1093/jxb/ert271 (PMC3808333; doi:10.1093/jxb/ert271)
Supplement: Supplementary Data [file supp_ert271_ert271_Supplementary_Data_amended_24_7.pdf]

Journal of Experimental Botany

Overexpressed TPX2 causes ectopic formation of microtubular arrays in the nuclei of acentrosomal plant cells.

Beáta Petrovská, Hana Jeřábková, Lucie Kohoutová, Věra Cenklová, Žaneta Pochylová, Zuzana Gelová, Gabriela Kočárová, Lenka Váchová, Michaela Kurejová, Eva Tomaštíková, and Pavla Binarová

Supplementary Table S1. ***In silico*** **analyses of AtTPX2 protein.**

Name	Positions (aa)	Description
**Domains:**		
**TPX2_importin**	313-487	domain pfam: PF12214
**TPX2**	649-705	domain pfam: PF06886
**Coiled-coil**	588-619	domain
**Motifs:**		
**Aurora**	20-29, 43-51	Aurora binding sites
**MTs**	220-463, 684-758	Microtubule binding sites
**NES**	499-507	nuclear export signal
**NLS**	228-236, 610-758	nuclear localization signals
**KEN box**	148-152, 275-279, 667-671	APCC binding destruction motifs
**D box**	327-645	APCC binding destruction motif
**Cyclin**	305-309, 332-336, 516-519	cyclin recognition sites
**FHA**	12-18,141-147, 218-224, 291-297, 373-379, 396-402, 437-443, 581-587	FHA phosphopeptide ligands
**MAD2**	361-369	binding motif
**MAPK**	41-47, 492-502, 538-546, 723-731	MAPK (Mitogen-activated protein kinase) docking motifs
**ProDKin**	53-59, 154-160, 271-277, 372-378, 419-425, 723-729	MAPK phosphorylation sites
**TRFH**	264-268	TRFH docking motif
**USP7**	5-9, 55-59, 95-99, 200-204, 289-293, 371-375, 396-400, 714-718, 748-752	USP7 binding motifs (deubiquitinating enzyme)
**HP1**	95-99	HP1 ligand (interact with the chromoshadow domain of Heterochromatin-binding protein 1)
**WW_Pin1**	53-58, 154-259, 271-276, 372-377, 723-728	WW domain ligands
**14-3-3**	180-186, 312-318, 395-400	14-3-3 ligands (interacts with specific phosphoserine and phosphothreonine containing motifs)
**COP1**	628-635	COP1 binding motif (Constitutive photomorphogenesis protein)
**EH1**	524-528	EH ligand (endocytotic processes)
**IQ**	294-312	Calmodulin binding IQ motif

Name	Positions (aa)	Description
**Motifs:**		
**SUMO**	68-71, 94-98, 208-211, 497-502, 529-532, 575-578	SUMO binding sites
**TRAF2**	4-7, 79-82	TRAF2 binding sites
**CK1**	8-14, 49-55, 98-107, 139-145, 400-406, 507-513, 747-753	CK1 phosphorylation sites (for Ser/Thr phosphorylation)
**CK2**	1-7, 33-39, 271-277, 289-295, 335-341, 435-441, 462-468, 491-497, 509-515, 580-586	CK2 phosphorylation sites (for Ser/Thr phosphorylation)
**GSK3**	1-8, 7-14, 8-15, 33-40, 49-56, 111-118, 115-122, 119-126, 132-139, 136-143, 200-207, 396-403, 395-404, 415-422, 431-438, 491-498, 714-721, 719-726, 732-739, 744-751	GSK3 phosphorylation recognition sites (for Ser/Thr phosphorylation)
**N-GLC**	132-137, 195-200	N-glycosylation sites
**PIKK**	98-104, 101-107, 195-201, 325-331, 580-586	PIKK phosphorylation sites
**PKA**	64-70, 122-128, 431-437, 491-497, 519-525, 694-700, 736-742, 742-748	PKA phosphorylation sites
**PKB**	674-682	PKB phosphorylation site
**PLK**	1-7, 5-11, 80-86	Site phosphorylated by the Polo-like-kinase
**ENDOCYTIC**	21-24, 543-546, 548-551, 570-573	Y-based sorting signals
**ER**	330-333, 429-432, 430-433	For ER localization

To predict putative interaction sites and binding motifs Pfam (Punta *et al.*, 2012), ELM (Dinkel *et al.*, 2012) and SUMOsp 2.0 (Ren et al., 2009) databases were used.

Supplementary Figure S1. Immunolocalization of AtTPX2 and actin in cell cultures of *Arabidopsis thaliana*

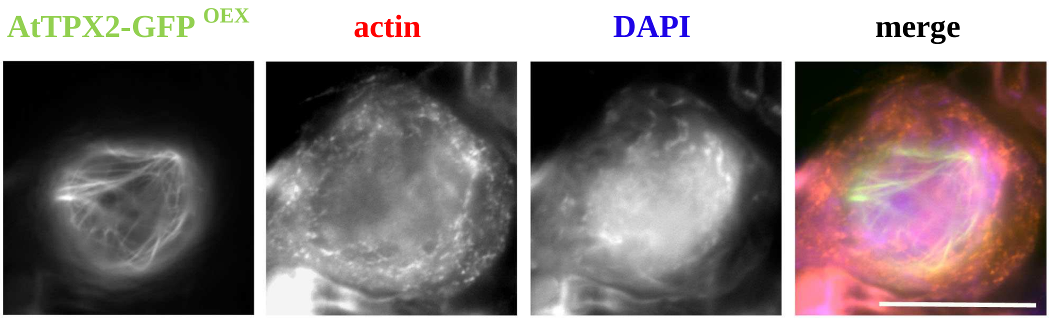

AtTPX2 fibres were not positive for actin immunolabelling. Bar: 10 µm.

Supplementary Figure S2. AtTPX2 decorated fibres were resistant to taxol in cell cultures of *Arabidopsis thaliana*

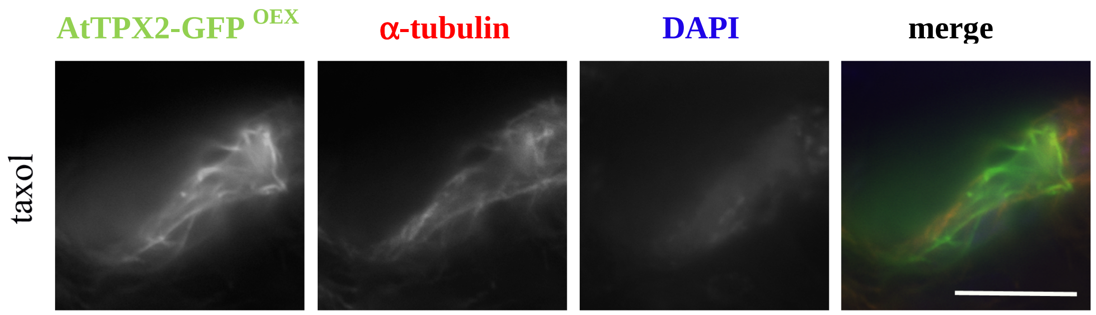

Further bundling of AtTPX2 decorated microtubules was not observed after treatment with 5 µM taxol for 3 hours. Bar: 10 µm.

Supplementary Figure S3. Immunofluorescence localization of Ran in AtTPX2-GFP overproducing cells.

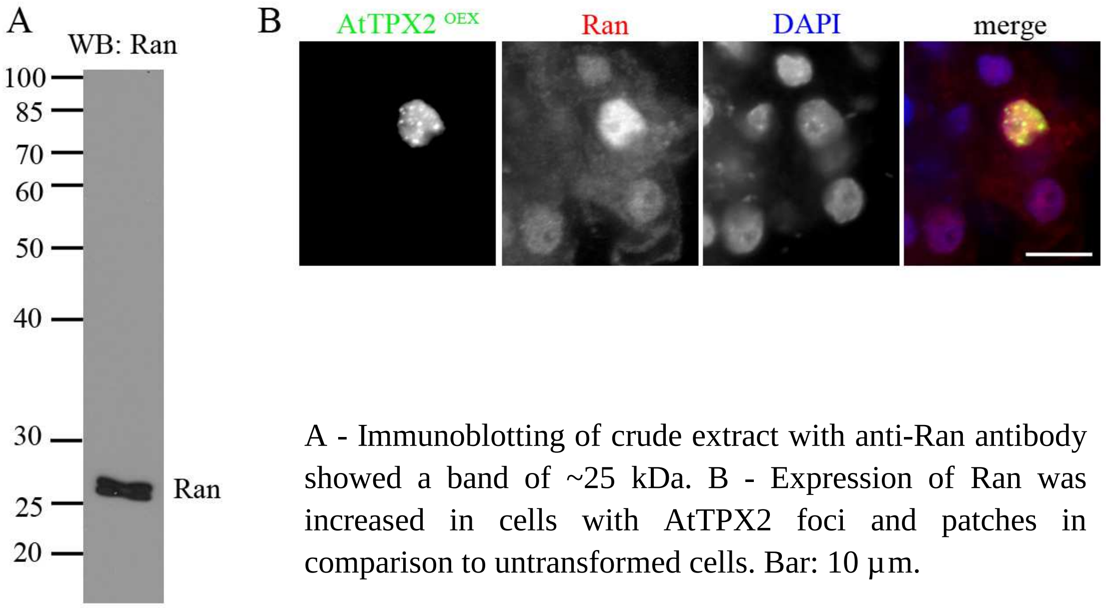

A - Immunoblotting of crude extract with anti-Ran antibody showed a band of ~25 kDa. B - Expression of Ran was increased in cells with AtTPX2 foci and patches in comparison to untransformed cells. Bar: 10 µm.

Supplementary Figure S4. Importin copurified with AtTPX2-GFP from *Arabidopsis* cultured cells.

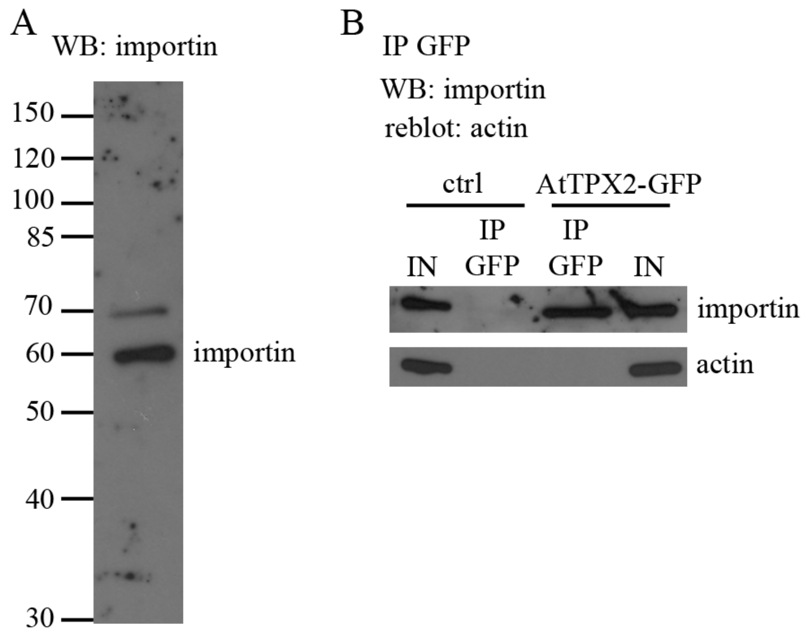

A - Immunoblotting of crude extract with anti-importin antibody showed a band of ~60 kDa. B - Immunoblotting with anti-importin antibody showed that importin was copurified with AtTPX2-GFP using GFP trap (IP GFP) from extract prepared from cell culture expressing AtTPX2-GFP. Untransformed wild type cell culture Ler was used as a negative control for GFP immunopurification. Immunoblotting with irrelevant antibody anti-actin was used as a second negative control.

Supplementary Figure S5. Colocalization analyses of AtTPX2 and importin in *Arabidopsis* cultured cells.

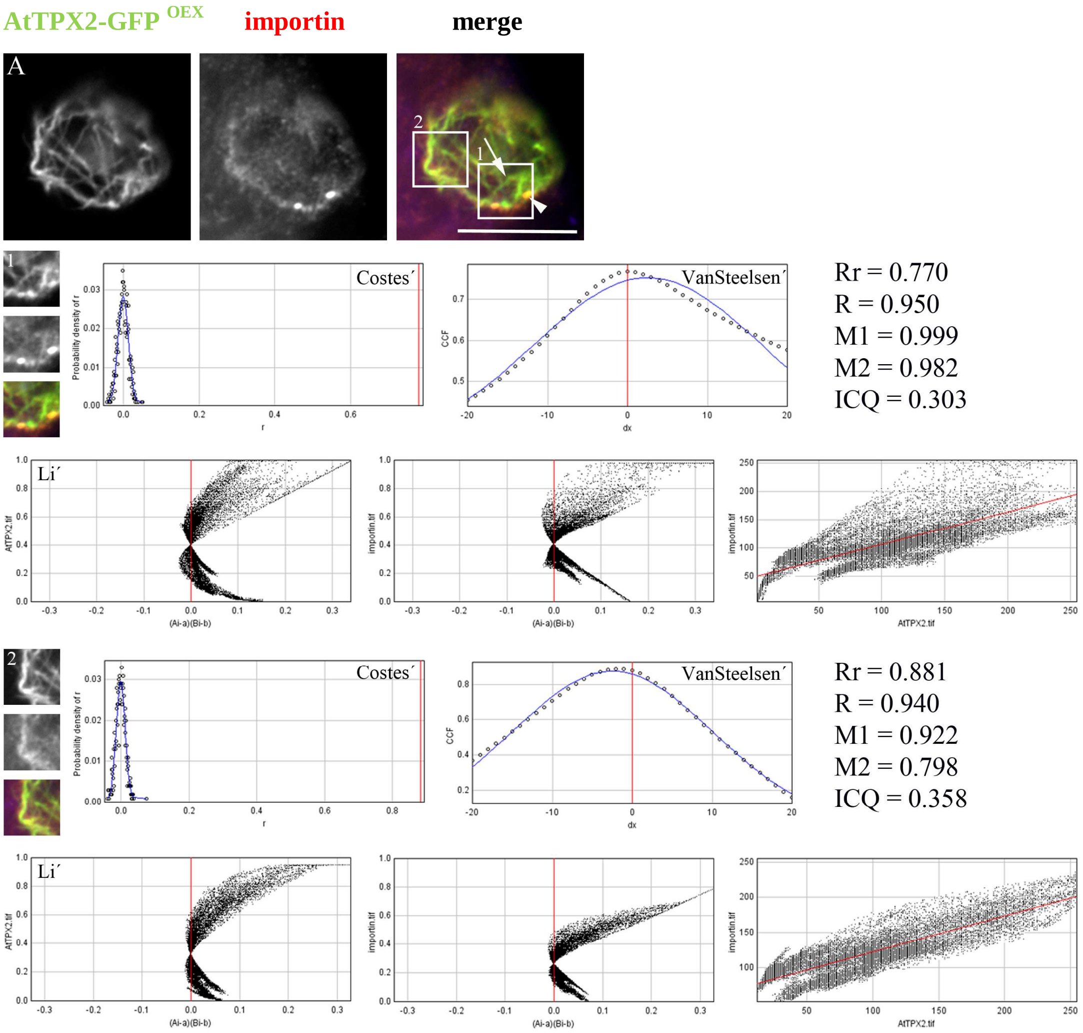

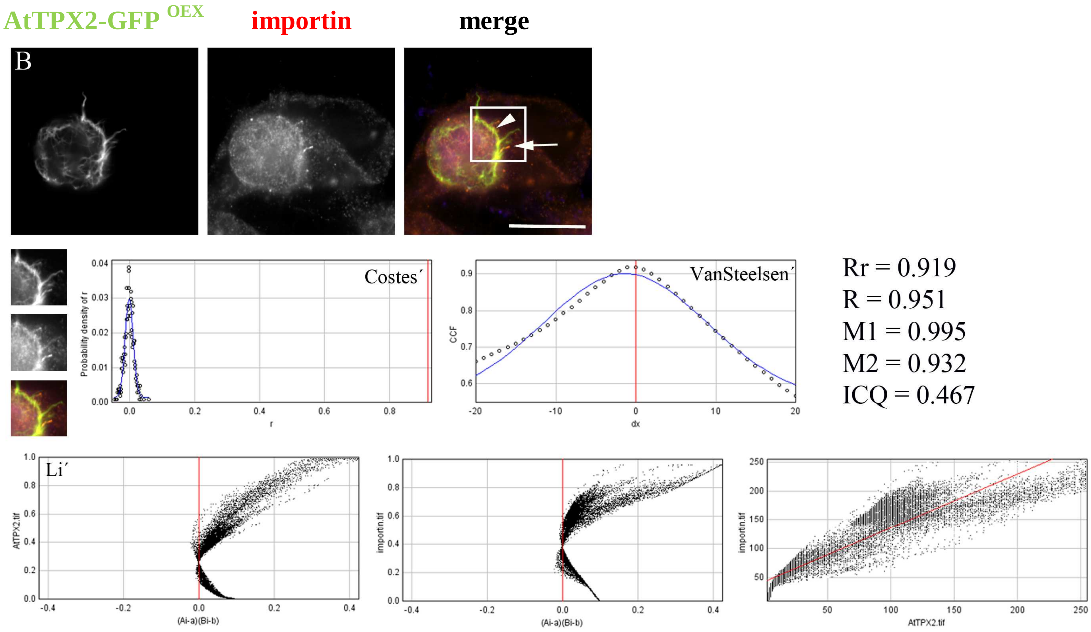

AtTPX2-GFP decorated fibres were present in close vicinity to the importin decorated nuclear envelope (A, B, arrowhead). Importin was present with AtTPX2 in dots on the nuclear envelope (A, B, arrowhead) and in the fibres (A, B, arrows). Pearson´s correlation coefficients (Rr) in Figure S8A (insets 1, 2) and S8B (0.770, 0.881, and 0.919, respectively) demonstrated colocalization of AtTPX2 and importin on the nuclear envelope and in the fibres in plant nuclei. However, Pearson´s coefficient in Figure S8A, inset 1 seemed to be complete colocalization with different intensities. The results of the Pearson´s correlation coefficient were confirmed with Manders´ coefficient (M1, M2). Figure S8 showed the completely colocalizing structure peak at the dx = 0 and bell-shaped curve. However, a difference in fluorescence intensity led to the reduction of the height of the bell-shaped curve, whereas the peak was still at dx = 0 (B). Non-colocalizing pixels in the Li´s approach are shown on the left side of the plots. The quantitative colocalization analyses of AtTPX2 and importin showed high degree of colocalization especially on the nuclear envelope. Colocalization analyses of AtTPX2 and importin was performed with ImageJ plugin JACoP (Bolte and Cordeliéres, 2006). Bars: 10 µm.

Supplementary Figure S6. AtAurora1-RFP copurified with AtTPX2-GFP from *Arabidopsis* cultured cells.

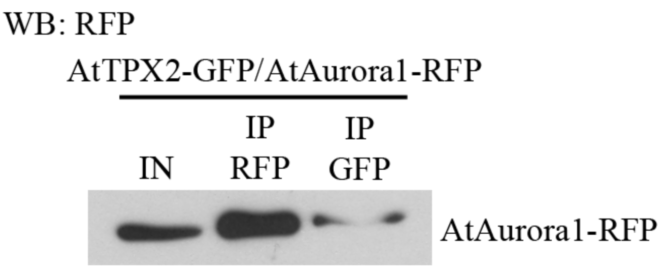

Immunoblotting with anti-RFP antibody showed that AtAurora1-RFP was copurified with AtTPX2-GFP using GFP trap (IP GFP) from extract prepared from cell culture co-expressing AtAurora1-RFP and AtTPX2-GFP. AtAurora1-RFP was purified by RFP trap (IP RFP).

Supplementary Figure S7. Overexpression of AtTPX2 and ΔN-AtTPX2 in *Arabidopsis* nuclei

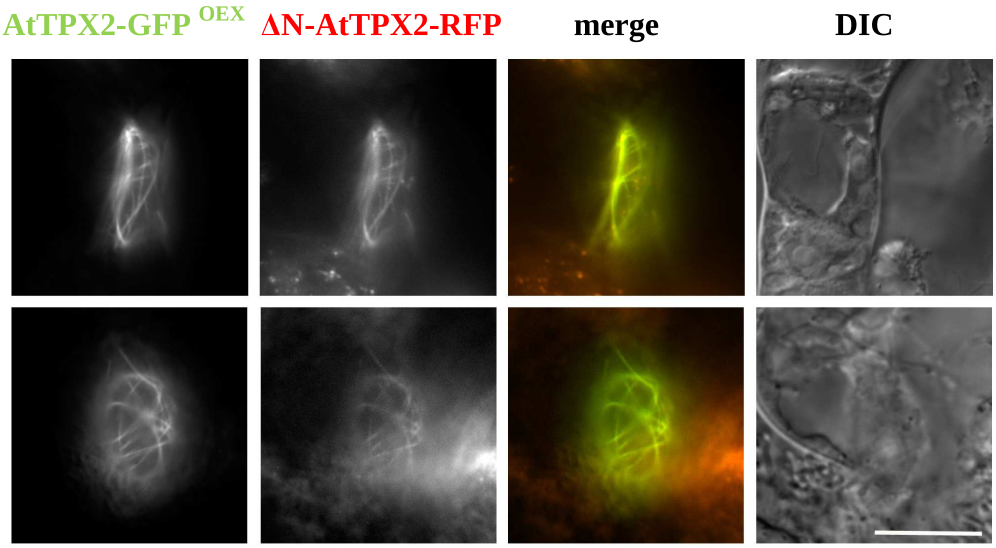

AtTPX2-GFP and its truncated version ΔN-AtTPX2-RFP localized together on microtubular array in the *Arabidopsis* nuclei. Bar: 10 µm.

Supplementary Figure S8. Treatment of mitotic microtubules with Aurora kinase inhibitor ZM447439 in cell cultures of *Arabidopsis thaliana*

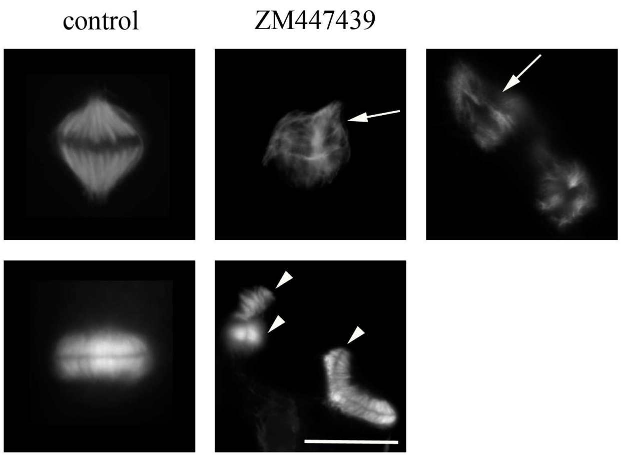

Microtubular arrays in the control (wild type) cells were affected after to the Aurora kinase inhibitor ZM447439 treatment. Multipolar mitotic spindle (arrows), fragmented phragmoplast (arrowheads) were often observed. Bar: 10 µm.

Supplementary Figure S9. Ectopic nuclear microtubular bundles were not affected by Roscovitine treatment in cell cultures of *Arabidopsis thaliana*

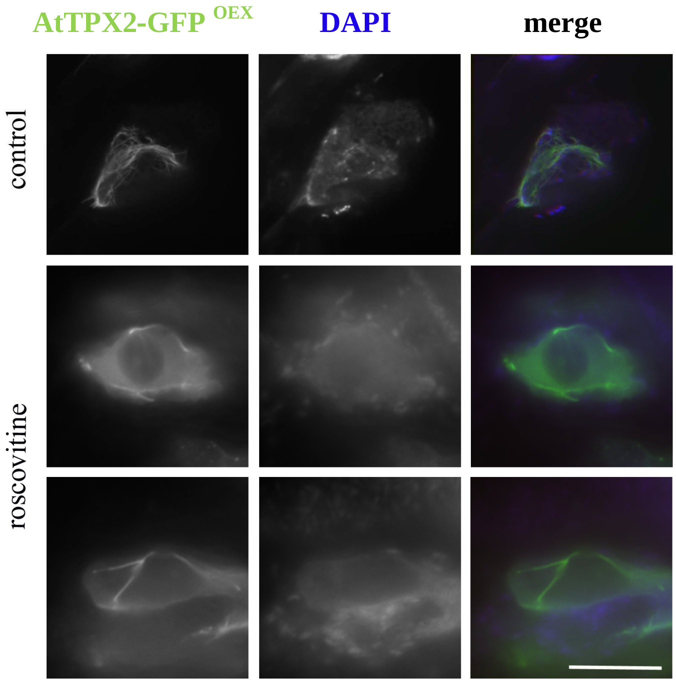

Formation of AtTPX2 decorated microtubular fibres was not affected with 100 µM Roscovitine, a cyclin dependent kinases inhibitor for 2.5 hours. Bar = 10 µm.

Supplementary Figure S10. Evans Blue viability test in *Arabidopsis* cell cultures with overproduced AtTPX2.

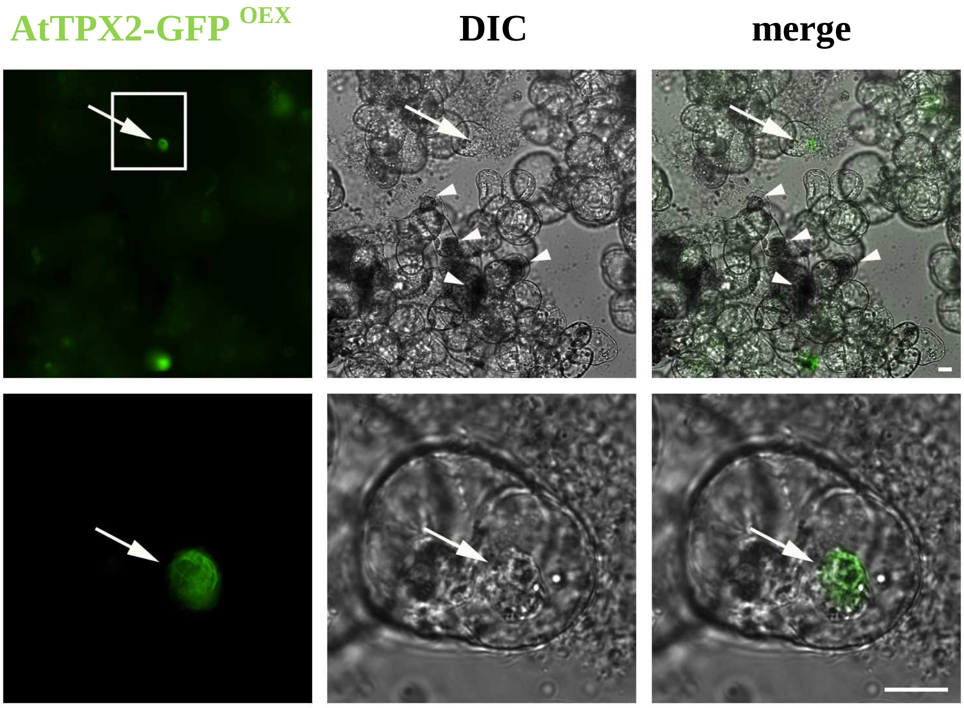

Evans Blue did not stain viable *Arabidopsis* cells with AtTPX2-GFP decorated fibres (arrows; n=68). Arrowheads showed death cells (dark grey colour). n = total number of AtTPX2-GFP analysed cells. Bars = 10 µm.
